# Alternative Treatments to Pharmacological Therapy in Pediatric Populations With Attention-Deficit/Hyperactivity Disorder (ADHD): A Scoping Review

**DOI:** 10.7759/cureus.55792

**Published:** 2024-03-08

**Authors:** Lexie Leon, Tram Tran, Meera Navadia, Janavi Patel, Annelies Vanderveen, Maria I Cruz, Thuy-Mai Le, Freda B Assuah, Victoria Prager, Darshil Patel, Joshua M Costin

**Affiliations:** 1 Dr. Kiran C. Patel College of Osteopathic Medicine, Nova Southeastern University, Fort Lauderdale, USA; 2 Osteopathic Medicine, Nova Southeastern University Dr. Kiran C. Patel College of Osteopathic Medicine, Clearwater, USA; 3 Medical Education, Nova Southeastern University Dr. Kiran C. Patel College of Allopathic Medicine, Fort Lauderdale, USA

**Keywords:** attention-deficit/hyperactivity disorder (adhd), complementary and alternative medicines, computer-based intervention, mindfulness-based therapy, music intervention, nerve stimulation, pediatric drug, pediatric nutrition, pediatric pharmacology, physical activity

## Abstract

In recent years, there has been an increase in the prevalence of the diagnosis of attention-deficit/hyperactivity disorder (ADHD), a cognitive and behavioral disorder in which individuals present with inattention and impulsivity, in the pediatric population. With an increase in diagnoses, there is also increasing concern regarding overdiagnosis and overtreatment with medications for ADHD. The objective of this study was to map out and compile the recent literature pertaining to alternative therapies (e.g., physical activity, diet, mindfulness, and computer-based interventions) for children and adolescents diagnosed with ADHD in an attempt to reduce or replace the use of pharmacological therapy. This scoping review searched articles from multiple databases (PubMed, ScienceDirect, Web of Science, Directory of Open Access Journals, Scopus, and CINAHL). Using search terms “children with ADHD,” “alternative treatment,” and “cognitive behavioral therapy,” articles were identified that were specific to the research question. The inclusion criteria were patients under the age of 18 with a previous diagnosis of ADHD, no other comorbid illnesses, alternative treatments, and was limited to studies published between 2012 and 2022. After removing duplicates, screening for eligibility criteria, and conducting a critical appraisal of the articles, 16 articles were retained for the final review. The main alternative therapeutic domains that emerged were (1) physical activity, (2) diet, (3) mindfulness, (4) computer-based interventions, and (5) miscellaneous interventions. Seven articles assessed the effect of physical activity on executive and cognitive function in children and adolescents with ADHD. Most findings showed improvement with increased physical activity. Two articles explored the effect of diet on the improvement of ADHD symptoms and reported a positive impact. The two articles that evaluated the effects of mindfulness on ADHD symptoms reported a reduction in ADHD symptoms. Two studies evaluated the use of computer-based interventions as an adjunct treatment in children and adolescents with ADHD; improvements in symptoms were reported. One study each evaluated interventions based on music and nerve stimulation. These showed an improvement in attention, memory, and executive function. With the increasing prevalence of ADHD diagnosis in children and adolescents, alternative and/or adjunctive treatments may be a viable and valuable alternative to pharmaceutical interventions. The findings from this review suggest that multiple non-pharmacological interventions effectively reduce symptoms of ADHD in children and adolescents, including diet, exercise, mindfulness, computer-based interventions, music, and nerve stimulation. While there are implications for alternatives to be used in the future, more research is warranted using larger samples with controlled trials.

## Introduction and background

Attention-deficit/hyperactivity disorder (ADHD) is a neurodevelopmental disorder in which individuals present with a range of symptoms, including inattention, hyperactivity, and impulsivity [1]. The Centers for Disease Control and Prevention (CDC) reports that 3.3 million children and adolescents in the United States between the ages of 12 to 17 years were diagnosed with ADHD from 2016 to 2019 [2].

In children and adolescents, ADHD can negatively affect their schoolwork as well as relationships with peers and family [1]. The CDC recommends a combination of parent education, adjustments in the school, and either stimulant or non-stimulant medications to help manage this condition [3]. After the identification of ADHD by the American Psychiatric Association, numerous medications were quickly developed. However, some of the recommended medications can have adverse effects on children and adolescents. Methylphenidate and amphetamine, for example, are stimulant drugs approved by the United States Food and Drug Administration (FDA) for the treatment of ADHD in children and adolescents. They work by increasing dopamine and norepinephrine in the brain [4]. Potential side effects include reduced appetite, insomnia, and, rarely, psychotic disorders, cardiac issues, and death [5]. In addition, serotonin and norepinephrine reuptake inhibitors (SNRIs) are non-stimulant drugs used in the treatment of ADHD [6]. They increase the amount of serotonin and norepinephrine in the brain and carry an increased risk of suicide attempts [6]. Not all patients respond to these medications with optimum results, and they are contraindicated in some patients with certain pre-existing conditions. There are concerns among some parents regarding overmedicating their children while others lack proper access to healthcare and are not able to access medication even if they would like to. As such, it is important to explore alternative treatments for ADHD.

This scoping review assesses gaps in the literature pertaining to alternative treatments for ADHD in children and adolescents. The alternative treatments being investigated include physical activity, which is thought to naturally increase dopamine levels in the brain [7]; a healthy diet which is widely thought to be associated with optimum brain function [8]; mindfulness which helps train children and adolescents how to regulate their emotions and focus their attention [9]; computer-based interventions, involving games that engage the patient that are thought to increase executive functioning through cognitive training [10]; music therapy which is thought to work by increasing serotonin in the brain to improve their stress-coping abilities [11]; and direct nerve stimulation which is thought to make long-term changes to the excitability of stimulated neurons [12]. With this in mind, this scoping review asks the question, “Are there efficacious alternative or adjunctive treatments for ADHD in children and adolescents?”

## Review

Methodology

Eligibility Criteria

The present systematic review adheres to the current Preferred Reporting Items for Systematic Reviews and Meta-Analysis guidelines. The articles included in this scoping review had to include subjects that were under the age of 18 years old with diagnosed ADHD and include the effects of a certain treatment on the symptoms of ADHD. Initially, articles published outside of the United States were excluded; however, after the initial review of article titles and abstracts, the search was expanded to other countries, as articles published in the United States on the topic that met the study’s inclusion criteria were limited. Reports from the CDC and FDA were also included to obtain statistics about the prevalence of ADHD in children in the United States and current treatment recommendations.

Articles were limited to recent articles published between 2013 and 2023 to review the most updated and validated studies. To understand and efficiently utilize the resource, only articles written in English were used. In addition, review articles were not allowed to be used for writing this research study. Articles that included children with comorbidities and confounding illnesses and diseases were also excluded to strengthen the accuracy and validation of the study. Exclusion criteria included study designs that discussed dual therapies such as alternative therapies, in addition to medication due to the potential for confounding variables.

Search Strategy

The research question was based on the PCC framework, with the Population being children/adolescents with ADHD, the Concept being alternative treatments to medication for ADHD, and the Context being interventions for children with ADHD. Based on those definitions, the review question was “What alternative or adjunctive treatments are used for children and adolescents with attention-deficit/hyperactivity disorder in the United States?” Data collection took place on September 25, 2022, and searches were carried out in the United States National Library of Medicine National Institutes of Health (PubMed), ScienceDirect, Directory of Open Access Journals, Scopus, CINHAL, and Web of Science. Additional searches were conducted in specific journals, such as the Cochrane Database of Systematic Reviews, the Journal of Developmental and Behavioral Pediatrics, the Journal of Sport and Exercise Psychology, and the Journal of the American Academy of Child & Adolescent Psychiatry. These databases were used because ADHD is a cognitive behavioral disorder that has symptoms of inattention, hyperactivity, and impulsivity. The disease is most commonly diagnosed and treated by pediatricians and relevant research is, therefore, likely to be published in journals focusing on Pediatrics and Adolescent Psychiatry.

The research team used controlled descriptors from PubMed, ScienceDirect, Web of Science, Directory of Open Access Journals, Scopus, and CINHAL Headings and Health Sciences Descriptors. The following phrases were searched: (Alternative treatments for children with Attention Deficit Hyperactivity Disorder), (Non-pharmacological therapy for children with Attention Deficit Hyperactivity Disorder), (Exercise and Attention Deficit Hyperactivity Disorder), (Cognitive Behavioral Therapy for children with Attention Deficit Hyperactivity Disorder), (Diet and Attention Deficit Hyperactivity Disorder), and (Nutritional Therapy for children with Attention Deficit Hyperactivity Disorder).

In this review, studies included were on the subject of ADHD that examined alternative approaches, besides medications, to treat ADHD in children. Studies needed to be published in or translated to English and published within the last 10 years. Publications such as case studies, clinical trials, meta-analyses, journal articles, and randomized control trials were included in the search for alternative therapies for ADHD. Exclusion criteria included study designs that discussed dual therapies such as alternative therapies in addition to medication due to the potential for confounding variables.

Selection of Sources 

Each of the 10 members individually rated the initial search by reviewing the title and abstract. There were no journals that were 100% agreed on as yes or no by all authors. Therefore, the first and second authors went through each of the journals and discussed whether they should be included or excluded. The third author then resolved any disagreements.

Data Charting Process

Data were collected by following the pertinent inclusion/exclusion criteria initially set by the reviewers. This included parameters such as alternative treatments in children and adolescents, studies published within the past 10 years, the type of studies, and so forth. The three reviewers sorted through each article independently and charted the data as they saw fit in Excel. They then came together to collaborate on their results and made any changes to discrepancies they had between their iterative process.

Results

Overview

**Figure 1:**
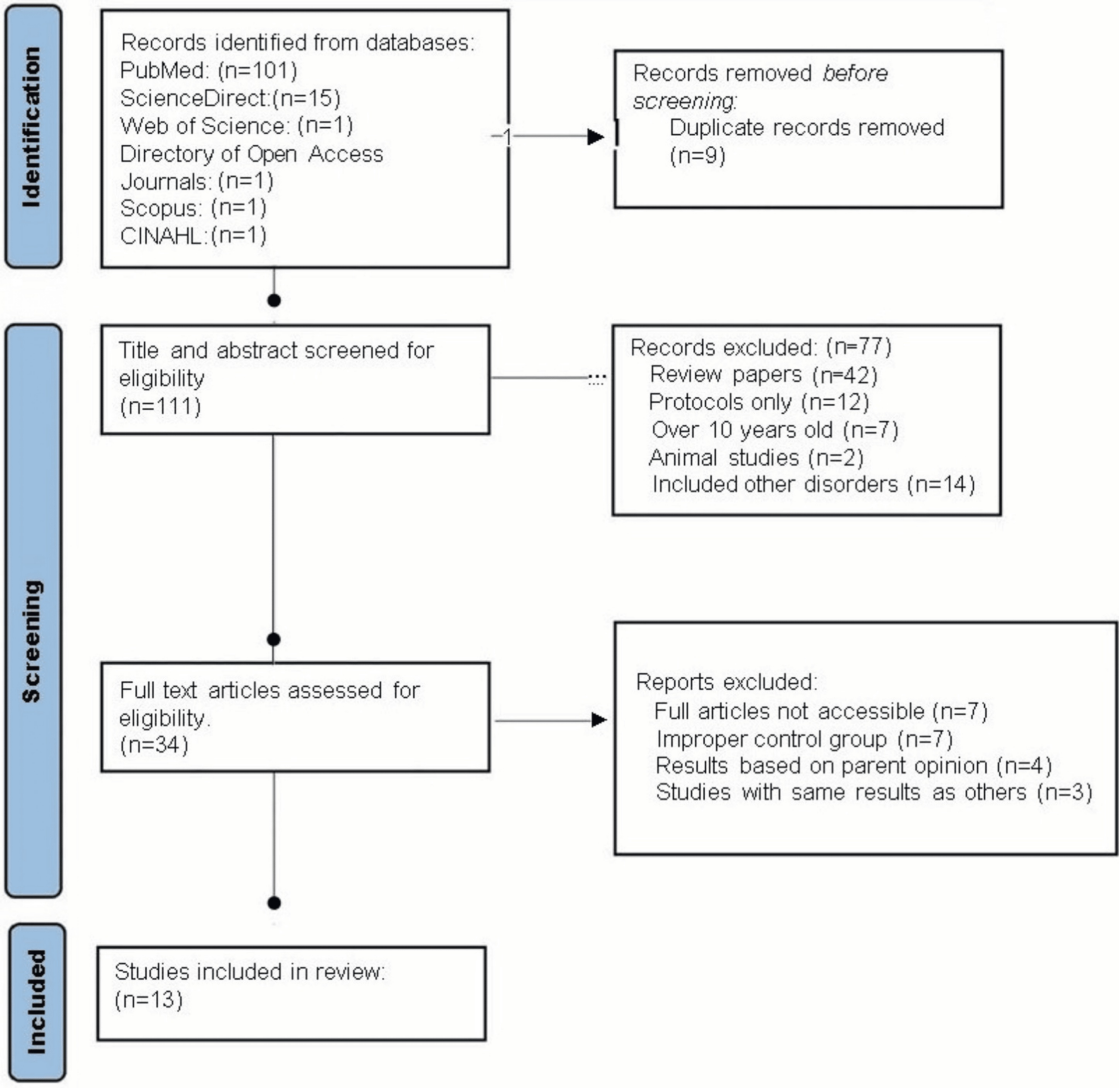
PRISMA chart for screening articles in the review. PRISMA: Preferred Reporting Items for Systematic Reviews and Meta-Analysis

A total of 13 articles met the study search criteria (Figure 1). Of the included articles, four focused on exercise interventions, three on diet interventions, two on mindfulness interventions, two on computer-based interventions, one on the effect of musicotherapy, and one on trigeminal nerve stimulation. Study designs in the included articles consisted of a journal article, a randomized controlled trial, a clinical trial, a randomized clinical trial, or a randomized triple-blind clinical trial. Table 1 contains a summary of the study design, data collection, study aims, findings, recommendations, and limitations of each article.

**Table 1:** A summary of the 13 articles included in the review.

Reference	Study design	Data collection	Study aim	Findings	Recommendations	Limitations
Chang et al., 2022 [13]	Randomized clinical trial	N = 48 children with attention-deficit/hyperactivity disorder (ADHD) (from grades 1 to 6)	The aim of this study is to discover if executive functions as well as handwriting problems would potentially result in improvement following training coordination of the arm, head, and eyes through playing table tennis, which entails visual concentration, in children with ADHD	Based on the results, both intervention groups demonstrated significant improvements not only on the Stroop Color-Word Test but in response time, handwriting, and the required time to achieve automation as well. However, the actual table tennis training group was the only group that achieved significant improvement on the Wisconsin Card Sorting Test. Overall, children with ADHD who played table tennis showed improvements in handwriting and executive functions in the short term	Look into whether the changes in executive function skills will last longer than 12 weeks, and if it could result in needing fewer stimulant medications	More participants were boys than girls. Limited range of ages
Liang et al., 2022 [14]	Randomized control trial	N = 80 children 6-12 years old (40 with ADHD, 40 without)	To study the effects of exercise on sleep and executive function in children with ADHD	Aerobic and neurocognitive exercise improves executive function and sleep quality in children with ADHD	Repeat the study with a larger population, including more with equal numbers of girls and boys. Repeat more assessments in the future to assess how long the effects last	A majority of the participants were male. The sleep quality study was subjective
Gawrilow et al., 2016 [15]	Journal article	Daily diaries for 10 consecutive days	Determine if exercise has an effect on executive functioning in children with ADHD	Physical activity does improve executive functioning in children with ADHD when compared to sedentary children with ADHD. Improvement began after 5 minutes of physical activity	Find a way to record the data not based on self-opinion	Findings were subjective
Nejati et al., 2021 [16]	Randomized control trial	N = 29 children, aged 7-12 years (15 with ADHD, 14 without)	To assess the impact cognitive rehabilitation has on children with ADHD by studying balance-cognitive training	The intervention had positive impacts on executive functioning in both ADHD and non-ADHD children	Repeat the study with control and experimental groups performing exercises of comparable intensity and a larger population	Control and experimental groups did not perform the same exercises
Motaharifard et al., 2019 [17]	Randomized triple-blind clinical trial	N = 50 children aged 6-14 years diagnosed with ADHD were randomly assigned to receive either methylphenidate or sweet almond syrup	This study evaluates sweet almond syrup, as a natural medication option, for ADHD in children	Results showed that methylphenidate and sweet almond syrup both showed improved symptom reduction in children with ADHD	Sweet almond syrup can potentially be a complementary and alternative medication option in the treatment of ADHD	The study had a relatively small sample size. Additionally, further trials with larger sample sizes, various drug dosages, and longer treatment and follow-up should be conducted
Ríos-Hernández et al., 2017 [18]	Journal article	N = 120 children and adolescents aged 6-16 years. 60 newly diagnosed with ADHD Diagnostic and Statistical Manual of Mental Disorders IV (DSM-IV) and 60 age and sex-matched	The study aims to find the relationship between adherence to a Mediterranean diet and ADHD diagnosis	The study found a correlation between people who did not adhere to a Mediterranean diet and ADHD diagnosis	More attention should be given to the diet of children with ADHD as these children are more likely to be on a bad diet, and improving their diet can improve their overall health as well	The case-control study design did not allow for developing a cause-and-effect relationship. Additionally, the dietary instruments used in the study were vulnerable to systematic and random errors
Bos et al., 2015 [19]	Randomized controlled trial	N = 40 male adolescents aged 8-14 years diagnosed with DSM-IV ADHD	The study aims to explore how omega-3 fatty acid supplementation impacts ADHD symptoms in adolescent males	Omega-3 supplementation helped to improve inattention symptoms in male adolescents who both have and don’t have ADHD	Compare omega-3 supplementation usefulness independent of medication	Quality of samples and sample size of the study
Huguet et al., 2019 [20]	Randomized controlled trial	N = 72 children, aged 7-12 years with and without ADHD	The study was conducted to investigate if a mindfulness intervention group could improve emotional deficiencies in children with ADHD	Regarding the therapy provided, the group with ADHD exhibited that they were able to regulate their emotions more efficiently	Allow teachers to submit the academic reports of the children in addition to the parents	Deficient emotional self-regulation is difficult to conceptualize
Clark et al., 2020 [21]	Clinical trial	N = 34 8-12 years old with ADHD	The study aims to look at the effects of Tai-Chi as a treatment inversion for children with ADHD and using a variety of measures to observe the impact of the treatment on symptoms	The results showed a significant improvement for the post-treatment group in motor control	The study shows more support in evidence of the efficacy of mindfulness interventions for children with ADHD, and more specifically the symptoms related to motor performance	There was a lack of a control group to use as a comparison to the experimental group. Additionally, there was no randomization due to the small sample size
Meyer et al., 2020 [22]	Clinical trial	N = 40 children with ADHD, aged 8 to 11 years	The study aims to explore if inhibitory control (IC) using a modified stop-signal task training is a potential intervention target	Results showed that the treatment group for adaptive IC training games showed marked improvement in inattention and behavioral symptoms in comparison to the control group	Continued studies for this treatment are recommended, but so far promising results are shown	Small sample size
Steiner et al., 2014 [23]	Randomized controlled trial	N = 104 neurofeedback (NF) (N= 34), cognitive training (CT) n = 34	This study assesses two different computer attention training systems. The systems were administered during school to children who had a diagnosis of ADHD and compared with a control	Children who received NF showed significant improvement whereas children who received CT showed no improvement	NF showed significant improvements in ADHD symptoms compared to the control and CT groups. It is recommended that NF be used as a treatment intervention in children with ADHD	The study did not achieve as large of a sample size as first desired. Additionally, the researchers admit that more independent and thorough standards could have been used to confirm the diagnosis of ADHD in the children involved in the study
Zhu et al., 2022 [24]	Randomized controlled trial	N = 120 children, 79 males, 41 females, aged 2-7 (diagnosed with DSM-IV ADHD)	The study assesses the impact of musicotherapy combined with cognitive behavioral intervention on the executive function of children with ADHD and provides some reference for ADHD rehabilitation	The intervention group showed significant improvements in the indexes than those of the control group after the intervention (p < 0.05)	Musicotherapy combined with cognitive behavioral intervention has clinical application to improve the cognitive functions of children with ADHD	The study ignored analyzing the limitations of the study
Loo et al., 2021 [25]	Randomized controlled trial	N = 34 male children, aged 8-12 (diagnosed with DSM-5 ADHD)	The study aims to determine the effectiveness of cognitive and electroencephalogram (EEG) predictors from trigeminal nerve simulation (TNS) for ADHD	Executive functions and ADHD symptoms were reduced in children with ADHD who underwent TNS	Research is required regarding maintaining the impacts of TNS and interaction with other treatments	The study was limited in sample size, and therefore, both type 1 and type 2 errors could be prevalent

Physical Activity

A total of seven articles evaluated the effects of physical activity on executive or cognitive function in children and adolescents with ADHD, with the majority of findings showing improvements with increased physical activity [13]. Specifically, one study evaluated the effect of table tennis, which focuses on hand-eye-arm coordination and visual concentration, on executive function and school-based handwriting [13]. Results showed an improvement in executive function and handwriting problems in children with ADHD. Another study examined the combined effects of physical activity on executive function and sleep quality, which showed an improvement in both [14]. In addition to executive function, another study analyzed the impact of physical activity on affect, with results showing an association between lack of physical activity and a more depressed affect [15]. Participants in the physical activity group displayed improvement in executive functioning after five minutes of vigorous exercise compared to the control group which showed no improvement [15]. Other studies that analyzed the effects of generic physical activity on executive function showed improvements in various measures of executive control compared to control groups [16]. While most articles showed improved outcomes with the addition of physical activity, one of the articles highlighted the fact that physical activity is not as effective as medications such as methylphenidate in improving cognitive functioning [14].

Diet

According to three included articles, the diet impacts ADHD in children and adolescents [17-19]. While diet may not be a direct cause of ADHD, there is evidence that it may contribute to the onset of ADHD via high glycemic index foods that may contribute to the increased onset. Conversely, adherence to a Mediterranean diet with a generally lower intake of sugar is associated with a decrease in the diagnosis of ADHD [18].

Two studies concluded that nutritional interventions could be used as adjunctive therapy after the diagnosis of ADHD. In the first, the administration of a sweet almond syrup found in Ayurvedic medicine may reduce symptoms of ADHD as effectively as stimulant medications [17]. Of note, sweet almonds refers to the variety of almonds and not a sweetened syrup made from almonds. Omega-3 supplementation, through a diet containing 10 g of margarine daily with either eicosatetraenoic acid (EPA)/docosahexaenoic acid (DHA), has also been shown to improve symptoms in individuals who have ADHD [19].

Mindfulness

Two articles reported the effect of mindfulness on children with ADHD [20,21]. Both articles demonstrated that mindfulness therapy showed a significant reduction in symptoms of inattention and hyperactivity. One of the articles examined the effects of structured mindfulness cognitive therapy which showed improved dysfunctional emotion regulation in children with ADHD [20]. The second article demonstrated the effects of Tai Chi on improving motor system dysfunction in children with ADHD [21]. The study observed a correlation between motor changes and ADHD symptom changes, with improvement in motor symptoms coinciding with a reduction in ADHD symptoms across the treatment groups.

Computer-Based Interventions

Two studies examined the effects of computer-based interventions, which utilize technology to aid in the treatment of ADHD in children. The computer-based intervention centered around stop-signal task (SST) training which targets inhibitory control (IC) that is deficient in children with ADHD. Three different gaming programs were utilized [22]. The results of the clinical trial were inconclusive; while parents reported the training did help with impulse control, teachers reported no such reduction [22]. Computerized training of IC in children and adolescents diagnosed with ADHD could potentially be an option for adjunct treatment but necessitates further research [22].

Neurofeedback utilizes characters on a screen to represent brainwaves to teach patients how to alter their brainwaves to maintain their attention [23]. Meanwhile, cognitive training uses computer feedback, not brainwave feedback, to reinforce correct responses to attention-training exercises [23]. In the neurofeedback and cognitive therapy trial, significant improvements in ADHD using neurofeedback were found, but no improvements using cognitive therapy [23].

Musical Therapy

The single included article by Zhu et al. (2022) examined the effect of musical therapy on the cognitive function of children with ADHD [24]. A randomized controlled trial compared a group of children receiving musicotherapy with cognitive behavioral intervention to one receiving only music therapy. The study found that using music clues and focusing on musical rhythms aided in improving cognitive function in many of the domains tested, including attention and memory [24].

Nerve Stimulation

One included article examined the effects of stimulating the trigeminal nerve (i.e., cranial nerve five) and its effects on children and adolescents with ADHD [25]. Stimulation of the nerve was conducted by neurostimulator electrodes placed on the patients’ forehead every night. A randomized, double-blind trial measured the brain waves of the children and adolescents who underwent nerve stimulation and demonstrated that there was an improvement in executive function in the treatment group [25].

Discussion

There are various potential alternative or adjunctive treatments for ADHD such as physical activity, diet, mindfulness, computer-based interventions, musical therapy, and nerve stimulation. Physical activity and mindfulness interventions can be considered the most promising alternative treatments among all the options listed. Both methods showed significant improvements in ADHD symptoms: physical activity notably enhances concentration and executive functioning, while mindfulness helps improve emotional regulation, symptoms of hyperactivity, and impulsivity. Physical activity and mindfulness may also be used as adjunctive therapies to medication use. With significant improvements seen following the use of physical activity and mindfulness in executive functioning, concentration, and emotional regulation, it is thought that introducing these activities into the daily lives of adolescents with ADHD can decrease dependency on medication. Encouraging these activities with the concurrent use of medication emphasizes the importance of steering away from medication dependence and the idea that medication is the only effective method to treat ADHD. Physical activity improves executive functioning while mindfulness was more associated with improved emotional regulation. As each activity is directed at different aspects of ADHD symptoms, further research should be done to investigate the additive effects of introducing both physical activity and mindfulness into the daily lives of children and adolescents with ADHD. While computer-based interventions and diet showed some improvement in ADHD symptoms, the study sizes were small, and the effects were not as significant as seen with physical exercise and mindfulness. Concerning diet, some studies emphasize the use of nutritional supplements while others emphasize the role of adjusting the entire diet. Further research needs to be done into which aspects of diet modification are most effective for ADHD symptom reduction before recommending it as an alternative to or adjunctive treatment option with medication.

While some alternative treatments are shown to be more effective than others, additional research is needed to investigate the additive effects of using the alternative treatments concurrently. For example, if physical activity, mindfulness, and diet modification show increased effectiveness when used together, it may be a better option than simply introducing physical activity in addition to medication. One of the main concerns with ADHD is the growing dependence on medication use. Further studies should examine the effectiveness of introducing alternative treatments at younger ages as opposed to introducing alternative treatments after adolescents have been on medications. Studies should be conducted to analyze the effect of introducing alternative treatments when children are initially diagnosed with ADHD or when children show early signs of ADHD before medication use.

Children and adolescents with ADHD can benefit from moderate-to-vigorous-intensity physical activity daily as demonstrated by improvements in cognitive and executive functioning [13]. Physical activity interventions sought to improve hand-eye-arm coordination, visual concentration, effect, and executive control through playing table tennis and five minutes of vigorous exercise. Results from these studies demonstrated an improvement in executive functioning and handwriting skills [13]. Additionally, physical activity was shown to improve sleep quality in children and adolescents with ADHD further improving cognitive functioning [14]. Physical activity can lead to an improvement in symptoms of ADHD as it can enhance concentration and executive functioning, which can improve focus and diminish impulsivity in children and adolescents with ADHD.

At this time, pharmacological therapy is considered the gold standard in treating ADHD in children as young as four years old, though current literature has suggested that children with ADHD can become dependent on ADHD medications. As such, physical activity can be incorporated as a viable adjunctive treatment method for children and adolescents with ADHD to limit medication usage, or potentially as an alternative treatment for those who wish to not use medication at all. This is a cost-effective and readily available alternative for many patients, though it may be less viable for certain populations such as those with certain disabilities.

Ríos-Hernández et al. [18] emphasized the correlation between adherence to the Mediterranean diet and ADHD diagnosis. The findings demonstrated that lower adherence to Mediterranean and unhealthy diet behaviors such as skipping breakfasts or eating fast food are associated with ADHD diagnosis. This study accentuates the role of a whole diet, rather than focusing on specific nutrients, in the treatment course of ADHD patients and may be a more affordable option for families that do not have insurance to cover traditional medication.

In the study by Sadat et al. [17], sweet almond syrup or methylphenidate was given to ADHD children and adolescents who participated in the research. This research is helpful and promising because some patients cannot tolerate methylphenidate, and having a diet alternative with comparable efficacy is necessary. The study showed that sweet almond syrup is a potential intervention for the future treatment of ADHD in children and adolescents. However, this is just one study, and follow-up studies are needed. Sweet almond syrup is an Ayurvedic medicine derived from the sweet almond (*Prunus dulcis*, variety *dulcis*) as opposed to the bitter almond (*Prunus dulcis*, variety *amara*). Despite the name, sweet almond syrup does not contain added sugars and is consistent with adherence to the Mediterranean diet as many patients attempt to limit sugar consumption in ADHD.

Another study also showed the effectiveness of omega-3 supplementation through a diet which is 10 g of margarine daily in improving symptoms in individuals who have ADHD [19]. The rating scale for symptom improvement was solely based on parents rating the attention of their children and adolescents. In fact, there was no change or impact of EPA/DHA supplementation on fMRI measures of brain activity in the treatment group when compared with the placebo group [19]. However, the research still proposes a plausible augmentation that can be effective in ADHD medication regimens. It is important to consult a physician or dietitian before giving a child sweet almond syrup and/or margarine to ensure the maintenance of an overall healthy diet. Though promising, diet changes appear most promising as an adjunctive treatment more than an alternative treatment as not enough high-quality studies have been performed to show equivalence to medication. There may also be some additional challenges to utilizing these treatments that could include families in lower socioeconomic statuses that cannot afford healthy foods, children and adolescents with many food allergies, and extremely picky eaters.

In the study by Huguet et al. [20], children and adolescents with ADHD and children and adolescents without ADHD were taken through mindfulness therapy. They were evaluated before and after the therapy for deficiencies in emotional regulation, including attention, aggression, anxiety, and depression. They found that after the therapy, children and adolescents with ADHD had improved emotional regulation. This shows that mindfulness therapy could be an alternative treatment choice for children and adolescents with ADHD.

In the study by Clark et al. [21], Tai Chi had a positive effect on motor control and function symptoms in children and adolescents with ADHD. The Physical and Neurological Examination of Subtle Signs Score (PANESS), which was utilized to measure the symptoms, showed improved motor control in gait and balance. The findings demonstrated that motor functioning may be used as a marker to monitor clinical outcomes in children and adolescents with ADHD as they were correlated with symptoms of hyperactivity and impulsivity in ADHD [21].

Both articles regarding mindfulness intervention portrayed significant benefits in symptom improvement for children and adolescents with ADHD when using this form of alternative treatment [20,21]. Each article targeted a specific domain of symptoms in ADHD, such as emotional regulation or motor control, and viewed the effect of the mindfulness treatment on children and adolescents. From these articles, it is shown that mindfulness treatment may be beneficial in managing and monitoring the wide variety of symptoms that can present in children and adolescents with ADHD as either an alternative or adjunctive treatment. Some cons include finding mindfulness teachers willing to work with hyperactive children and adolescents, and the potential cost associated with the classes.

Using neurofeedback intervention has shown improvements in ADHD symptoms in children and adolescents [23]. In the study by Steiner et al., teachers and parents both recorded greater attentiveness and further progress of ADHD behaviors when using neurofeedback but did not see any improvements with cognitive attention training interventions [23]. One aspect of neurofeedback interventions found that parents of children and adolescents who used neurofeedback interventions were less likely to increase their child’s stimulant medication dosage [23]. While this study did not examine neurofeedback interventions as a replacement for stimulant use, it did provide groundwork on a possible start to reducing stimulant medication for children and adolescents diagnosed with ADHD. Some limitations of this study included having a small sample size as well as not blinding the parents as to which group their child was placed in, possibly introducing bias [23]. Neurofeedback appears to be useful as an adjunctive therapy only for now, as there is no data on using it as an alternative treatment. Further research into neurofeedback interventions could potentially provide further medication relief for children and adolescents with ADHD.

While there seemed to be an improvement in inhibitory control (IC) when stop-signal task (SST) training was used, there was not enough evidence to prove that it effectively treated hyperactivity or inattention, which are some of the central symptoms of ADHD [22]. According to the teachers’ reports, SST training had no discernible influence on inattention [22]. However, this contrasted with parents’ reports of reduced symptoms of inattentiveness, though it is possible this was a result of parental bias or maybe teachers only seeing students while on medication at school [22]. Moreover, this study was conducted with a small sample size and needs to be replicated with a larger cohort and proper controls [22]. In addition to these findings, IC could be linked to theta oscillatory power through stimulation of the right inferior frontal gyrus [22]. Given that theta power is related to inattention symptoms, reducing theta power could lead to decreasing those symptoms [22]. Though targeting IC through SST training can potentially be used as a supplemental treatment for ADHD, there is currently not enough evidence to prove that it can be used to solely treat the aforementioned disorder. Hence, future studies should look into not only how to decrease potential biases while improving hyperactivity and inattention symptoms but also how to do so by learning more about how ADHD affects neural mechanisms, like IC.

Musical therapy combined with cognitive behavioral intervention was found to improve cognitive function and strengthen attention, key symptoms of ADHD [24]. The improvement of symptoms was found to be long-lasting, with no other side effects present in the children and adolescents from the treatment. Various cognitive tests demonstrated markedly improved scores in subjects throughout the trial, showing that this therapy can be significantly beneficial for children and adolescents with ADHD [24].

Music therapy is a new evolving form of therapy that may be useful for treating children and adolescents with ADHD. Many cognitive function improvements can result from the use of music therapy, especially in combination with cognitive behavioral intervention, another alternative treatment. The trial by Zhu et al. (2022) specifically examined children and adolescents that had no prior history of taking medication, allowing for a better understanding of the benefits of alternative treatment on its own [24]. This article examines the effects of treatment over a three-year time frame. A downside to this treatment is that music therapy may be expensive for some families, unlikely to be covered by insurance, and, due to its novelty, may be difficult to find a practitioner.

In the study conducted by Loo et al. (2021), the results showed improved cognitive measures in children and adolescents with the nerve stimulation treatment [25]. The treatment was shown to be significantly effective in children and adolescents with executive functioning weakness, though more studies need to be performed to bolster this claim. These are a set of skills used in everyday functioning and nerve stimulation demonstrates activation of the areas of the brain that control these functions. Although this treatment may become more popular in the future, it is still experimental, invasive, likely to be expensive, and difficult to find a practitioner.

Limitations

The search for this review was expanded beyond the United States because of minimal articles found initially. The lack of diversity in demographics and sample sizes was a limitation in many of the articles. Further trials would need to emphasize on a larger sample size. Some studies also lacked comparison to a control group. Additionally, there are varying factors that could contribute to the reprieve of ADHD symptoms such as changes in patient moods or external settings, and credit cannot fully be given to the respective alternative treatments. Finally, further research and more trials are required to understand the long-term benefits of alternative treatments for children with ADHD.

## Conclusions

ADHD is a prevalent condition among children and adolescents. Although there are currently accepted pharmacological approaches to treatment, these are often accompanied by adverse effects of varying severity or are not a preferred treatment by some parents. Therefore, it is necessary to explore viable non-pharmacological approaches to treatment. The findings from this review suggest that multiple non-pharmacological interventions effectively manage ADHD in children and adolescents, including diet, exercise, mindfulness, computer-based interventions, music therapy, and nerve stimulation. These interventions can be utilized alone in the treatment of ADHD or as an adjunct to existing pharmacological interventions. While these initial findings are promising, more research is needed to explore the strengths and limits of each technique and how to properly integrate such interventions into the patient’s care.
