# Correction: Alternative Treatments to Pharmacological Therapy in Pediatric Populations With Attention-Deficit/Hyperactivity Disorder (ADHD): A Scoping Review

**DOI:** 10.7759/cureus.c316

**Published:** 2025-09-24

**Authors:** Lexie Leon, Tram Tran, Meera Navadia, Janavi Patel, Annelies Vanderveen, Maria I Cruz, Thuy-Mai Le, Freda B Assuah, Victoria Prager, Darshil Patel, Joshua M Costin

**Affiliations:** 1 Dr. Kiran C. Patel College of Osteopathic Medicine, Nova Southeastern University, Fort Lauderdale, USA; 2 Osteopathic Medicine, Nova Southeastern University Dr. Kiran C. Patel College of Osteopathic Medicine, Clearwater, USA; 3 Medical Education, Nova Southeastern University Dr. Kiran C. Patel College of Allopathic Medicine, Fort Lauderdale, USA

This article has been corrected to fix the following typos and missing information in the first paragraph of the Results section:

Original version (incorrect): A total of 16 articles met the study search criteria (Figure 1). Of the included articles, seven focused on exercise interventions, three on diet interventions, two on mindfulness interventions, one on the effect of musicotherapy, and one on trigeminal nerve stimulation. 

Corrected version: A total of 13 articles met the study search criteria (Figure 1). Of the included articles, four focused on exercise interventions, three on diet interventions, two on mindfulness interventions, two on computer-based interventions, one on the effect of musicotherapy, and one on trigeminal nerve stimulation.

